# Oxygen in Leucæmia and Pseudo Leucæmia

**Published:** 1886-11

**Authors:** 


### *OXYGEN IN LEUCÆMIA AND PSEUDO LEUCÆMIA.*

M. Kinberger, in *Noveaux Remèdes*, relates the particulars of a case in which, arsenic having failed, the inhalations of oxygen brought about a rapid increase of strength with diminution of the hypertrophy of the spleen; at the same time the number of white globules were increasing and the red were returning to the normal proportion. At the expiration of several months the disease reappeared, but although the number of red globules had diminished those of the white were not increased. The inhalations of oxygen had effected a complete cure.

